# Encysted Tumors

**Published:** 1870-09

**Authors:** Frank H. Hamilton


### SELECTED ARTICLES.

### ARTICLE V.

### *Encysted Tumors.*

A lecture delivered at Bellevue Hospital Medical College.

By Prof. Frank H. Hamilton, M. D.

*Ranula; or Encysted Tumors of the Ducts of either the Submaxillary or Sublingual Glands—Syn. Batrachos; Frog-Tongue.*—The term ranula, from the Latin *rana*, has by some writers been restricted to obstructions of Wharton's duct, and by others to obstructions of the sublingual ducts. There is sufficient evidence, however, that a tumor answering to the description of a ranula, is found occasionally in the duct of the submaxillary gland, and sometimes, also, in the ducts of the sublingual. Serous cysts may also arise in the substance of either of these glands probably in their connective tissue, independent of their ducts. Tumors having some points of resemblance to a ranula may originate in obstructed muciperous glands. Finally, there are many examples of enlarged submaxillary bursæ, which very much resemble a ranula in their external appearance. It is proposed however, to limit the term to obstructions of the ducts of the submaxillary and sublingual glands. To the other forms of encysted tumors resembling ranula, the term "spurious," may be conveniently applied.

The differential diagnosis between these various forms, is usually attended with so many difficulties, that it seems proper to make a brief allusion to the anatomy and situation of the submaxillary and sublingual glands.

The submaxillary glands are situated below the inferior maxilla, in the anterior portions of submaxillary triangles. They are covered by the integument, the platysma myoides muscles and the deep cervical fascia. The duct of each gland, called the duct of Wharton, commences by numerous radicals, within the substance of the gland, forming a single tube of about two inches in length, and with walls much thinner than those of the parotid duct, terminating in a narrow orifice on the summit of a small papilla at the side of the frænum linguæ.

The sublingual glands, weighing usually about one dram; they are situated beneath the mucous membrane of the floor of the mouth, on each side of the frænum linguæ, reaching from the anterior margin of the submaxillary gland to the symphysis mentis. Their ducts, of which there are from eighteen to twenty for each gland, open into the mouth on the elevated crests of mucous membrane, caused by the projections of the glands on each side of the frænum.

While in some cases an exact differential diagnosis may be impossible, it is not difficult in many examples of tumors presenting the general appearance of ranula to determine the organ or structure to which they belong.

An obstructed submaxillary duct, in case the obstruction is at or near its origin, forms an ovoid, elastic, translucent swelling, first noticed beneath the tongue on one side of the frænum; and it does not press downward and give to the throat the peculiar fullness below the chin from which its name ranula is derived, until it has attained considerable size. Usually such tumors are not seen of a size larger than an almond; but in a few instances they have been known to enlarge to such a volume as to threaten suffocation. When opened, they are found to contain a perfectly clear, albuminous fluid, of the consistence of the white of an egg, with an alkaline reaction. They sometimes contain solid, phosphatic concretions; and in such cases the presence of these bodies may be recognized by the peculiar feel before the sac is opened.

When these tumors attain considerable size, they involve sometimes the whole length of Wharton's duct, including the canals leading into the gland; under which circumstances the gland occasionally undergoes absorption, in consequence of the pressure; and the tumor ceases to enlarge.

Obstructions of the sublingual ducts give rise to similar tumors, commencing first within the mouth, and subsequently pressing downwards, causing in some cases also, atrophy of the gland, and a consequent arrest of the growth of the tumor. The fluid found in the sublingual ducts differs from that secreted by the submaxillary gland only in being a little more viscid; but Rokitansky affirms that calculi occur in these ducts much more often than in the submaxillary. They are usually white and friable, varying in form and in size from a millet seed to a hazel nut.

Of both these forms of tumors there may be presented rare and exceptional examples, in which the obstruction taking place remote from the orifice, and perhaps near the glands, the pellucid oval projection beneath the tongue does not occur; but from an early period there is quite as much prominence caused by the tumor below the chin as in the floor of the mouth. When the tumor commences in the ducts within the gland itself, as I have seen happen several times, the floor of the mouth is only encroached upon at a late period. But their anatomical character may still be determined by the position of the tumors, and their previous history. I have sometimes been able to determine whether the ducts of Wharton were involved, by a careful examination of the orifices of the ducts, and by the salivary jet.

Those tumors which result from obstruction of the muciperous glands, are now circular in form, and probably never attain much size, or press downwards, into the neck. They contain an abundance of epithelial cells, degenerated epithelium, oil cells, and granulation cells, giving to the contents the consistence of oil or of putty. They are opaque, although covered only by mucous membrane, and in this regard differ from the two forms before mentioned.

Tumors formed from the connective tissue in this region are not translucent. They lie usually more deeply, and contain serum only, very slightly colored, and probably some phosphates or calcareous concretions.

Finally, tumors occasioned by enlargement of bursæ, lie deeper from the floor of the mouth, projecting less into its cavity, and much more below the chin. They contain a clear serum, or serum colored by more or less blood corpuscles. These are sometimes congenital.

It may be proper to call attention again to the fact, that I have enumerated two forms of true ranula; one in which the obstructions of the duct takes place at or near its orifice, and which is characterized by the translucent sublingual tumors, accompanied with more or less projection below the chin; and a second, in which the obstruction occurs at or near the gland, and there is no sublingual translucent tumors, but only a general uplifting of the floor of the mouth, and much greater prominence of the tumor below the chin.

There is also a third condition to which I called attention in a communication made to the GAZETTE in February, 1868, in which the obstructions occurring in any portion of the duct, is partial and temporary, and does not occasion, properly speaking an encysted tnmor, but a sudden dilatation of all the radicals situated within the gland, causing the gland to enlarge, especially during the act of mastication. In such cases, as in one example cited, an active cathartic may accomplish relief; or the flnid may be expressed by pressure upon the gland. If however, the obstruction is continued a sufficient length of time, the walls of the ducts yield at one point or other, and a cyst is formed.

*Treatment.*—The treatment of that form of true ranula caused by obstructions at or near the orifice of the ducts, consists in opening the sac, evacuating its contents and, maintaining or re-establishing the orifice.

It has been observed that the fistulous orifice thus made, cannot always be maintained if the sac is simply incised; but that it is often necessary to remove a large portion of the sac, so as to prevent the edges from falling into contact; and that a successful result is more certainly ensured by cauterizing the edges and interior of the sac, and then laying a bit of lint between the edges of the wound.

In case the obstruction is at or near the gland and the substance of the gland itself is involved in the tumor, as may be indicated by the fact that the tumor does not present the usual translucent appearance under the tongue, but seems to lie deep under the floor of the mouth, projecting chiefly beneath the chin, between it and the hyoid bone, then it will be more prudent to open the sac from the most salient point below the chin; and after the lapse of several days, when the sac has been thoroughly drained, to seek to destroy its secreting surface by injections of tincture of iodine, tents, etc. Opening the sac from the mouth under these circumstances, is attended with the hazard of hæmorrhage; and as it must be kept open a long time, it is exceedingly annoying to the patient; it is more difficult to maintain the fistulous orifice; and finally, it is not so likely to result in a complete obliteration of both the duct and the gland, which must be now the chief point to be attained.

Moreover, it is the form of ranula which is most likely to be confounded with simple serous cysts developed in the substance of the gland, or in the connective tissue adjacent, or with bursal tumors, in either of which latter cases complete destruction of the secreting surfaces and not the re-establishment of the original canal, is to be desired. The destruction of the cyst can be accomplished with the most certainty, through an external, free, and depending opening.

It is true, however, that in the case of simple cysts, here as elsewhere, it is preferable to remove the cyst entire by dissection; but the difficulty in any case of making an accurate diagnosis and the embarrassments which often attend the destruction of encysted tumors in this region, will justify the surgeon generally in resorting to incision rather than excision.

Finally, the treatment of these varions tumors, including ranula properly so called, and spurious ranula, may be summed up as follows:

In case of a well characterized translucent ranula, presenting itself almost exclusively within the mouth, an attempt must be made to re-establish the opening of the duct by free excision of the projecting portions.

In case of a deep-seated ranula, probably involving the gland, it is more prudent to open from the integument; and success is more certainly ensured by continual suppuration, causing thereby a destruction of both the duct and the remnant of the gland itself.

In case of a bursæ, the same course should be pursued as in the deep seated ranula.

And while in case of a simple serous cyst, formed either in the gland or the adjacent connective tissue, if the diagnosis were complete it might be proper to attempt its extirpation by dissection; yet considering the difficulty of diagnosis, the hazards and difficulties of the operation, and the generally successful results of incision followed by injections, the latter course must generally commend itself to the prudent surgeon; that is to say, the same treatment as for a deep-seated ranula, or a bursa.

*Encysted Tumors of the Parotid Gland and of its Ducts.*—The parotid gland, situated upon the side of the face in front of the ear, is the largest of all the salivary glands. Its excretory duct originates by numerous radicles, which unite within the substance of the gland to form a common duct, called the duct of Steno, which passes almost directly forwards over the massiter, penetrating the buccinator, and after coursing a short distance between the buccinator and mucous membrane, enters the buccal cavity opposite the second molar of the upper jaw. Its walls are dense, consisting of an outer, or fibrous coat, containing contractile fibres, and an inner or mucous coat, lined with columnar epithelium. The canal is about the size of a crow quill.

The secretion of the parotid is clear and watery in appearance, has a mucilaginous feel, it is alkaline in its reaction, and, among other ingredients, contains always a compound of sulpho-cyanogen.

Obstuctions of the duct of steno have not hitherto been classified as tumors; and probably for the reason that they cannot in any case be subjected properly to the some treat. ment which is applicable to other tumors; that is to say, they can never be properly treated by extirpation—the only proper surgical expedient is to attempt to re-establish the communication with the buccal cavity; but the same objections will hold good against recognizing obstruction of the ducts of the submaxillary and of the sublingual glands as tumors. I prefer to consider these obstructions in this connexion.

*Obstruction of the Duct of Steno at its Orifice.*—Occasionally as the result of abrasion and consequent adhesion of the mucous membrane, at the point where the duct opens into the mouth, a small pellucid tumor is formed under the mucous lining, which, after a time, opens spontaneously, and a cure is affected.

*Obstructions of the Duct of Steno in its continuity, from Adhesions caused by Wounds, Ulcerations, Gangrene, etc.*, causing in some cases a temporary elastic, oblong elevation, on the side of the face; but the saliva soon makes away for itself, through the unsound tissues, and causes a fistula upon the side of the face, which is exceedingly difficult to close. There is, perhaps, no better plan than to pass a pretty large silk seton through the orifice of the fistula into the mouth, tying its two ends upon the chin; after the lapse of two or three weeks, it may be removed, and the fistula closed externally, by freshening its edges and uniting them with silver sutures.

*Encysted Tumors from Calculi in the Duct of Steno.*—These tumors present themselves as hard, oblong nodules on the inside of the mouth; the presence of the calculus having sometimes caused inflammation and adhesion of the walls of the duct, so that the sac contains saliva and pus as well as the calculus.

These calculi are generally composed of the phosphate of lime, with a triple phosphate.

They must be laid open and the calculous removed from the inside of the mouth, so as to avoid a salivary fistula.

*Encysted Tumors Caused by Obstructions of the Ducts of the Labial, Buccal, Lingual, Pharyngeal Glands, and Follicles of the Mouth.*—*Anatomy of the Glands.*—The labial, belonging also to the system of salivary glands, are situated beneath the mucous membrane covering the orifice of the mouth, and especially the inner surface of the lips.

They are rounded in form, and of about the size of a small, pea, opening by minute orifices upon the surface of the mucous membrane.

The buccal glands, similar to the labial, but smaller, are situated between the mucous membrane and buccinator muscle, except two or three called molar glands, which lie between the masseter and buccinator; these latter are somewhat larger than the submucous buccal glands, and their ducts open opposite the last molar tooth.

There are also a few small glands of the same character under the mucous membrane of the posterior half of the hard palate; those of the under surface of the soft palate are larger and more numerous.

Those of the tongue (lingual) are chiefly on the posterior third of the dorsum, and a few are found along its edges, and at the tip.

In the pharynx, especially at its upper part, these glands are numerous.

In addition, the mucous membrane of the mouth is supplied with numerous simple and compound mucous follicles:

they extend over the entire surface of the tongue, but are most numerous at the posterior portion.

The tonsil, situated between the two pillars of the sôft palate is composed of lacunæ and closed vesicles, embedded in connective tissues and vessels. The secretion is a viscid mucus, designed to lubricate the food in its passage through the fauces.

*Diagnosis and Treatment.*—Encysted tumors of the labial glands are of frequent occurrence, presenting themselves as small, round, vesicular elevations, with semi-opaque walls, seldom attaining a greater size than a small pea, and containing a thin transparent, or nearly transparent muculent fluid.

They are often ruptured spontaneously, but in that case they generally reappear very soon.

Simple puncture and evacuation of the contents is generally followed by the same result.

Excision is therefore the only reliable resource. This may be practised by seizing the vesicle, with a small portion of the adjacent tissues, and excising the whole with a pair of scissors. In some cases I have found it necessary to remove the entire gland, the removal of the expanded duct being followed by a renewal of the cyst.

Very recently I have had occasion to remove a cyst formed in one of the buccal glands; but the remaining muciperous glands of the mouth and pharynx, seldom become sufficiently obstructed to cause tumors; or if they occur, it is probable that their walls easily give way, and a spontaneous cure is affected.

When they occur upon the tongue, as happens now and then, especially near the middle of the dorsum, it is not advisable to attempt their extirpation. A case was reported very recently in which an operation made for the removal of an encysted tumor of the tongue proved fatal They should be treated by incision and injection. Indeed this observation applies to all simple encysted tumors which may form in the mouth, and remote from the orifice.—*Medical Gazette.*

